# Bibliographical Norices

**Published:** 1840-04-01

**Authors:** 


### Periscope;

OR,

### CIRCUMSPECTIVE REVIEW.

"Ore trahit quodcunque potest, atque addit acervo."

### Notices of some New Works.

TRANSACTIONS OF THE MEDICO-BOTANICAL SOCIETY OF LONDON. Edited by *W. H. Judd,* Esq. Senior Secretary. 8vo, pp. 248, London, 1839.

THE present small volume of the Transactions of this useful Society contains several Papers which may interest the practical portion of our readers. We shall select the main facts that they present.

I. ON A NEW PREPARATION FROM THE PIPER CUBEBA. By W. H. JUDD, Esq. M.R.C.S., Surgeon in the Scots Fusileer Guards, and Fellow of the Medico-Botanical Society.

Mr. Judd is well known to have devoted much attention to the study and the treatment of venereal affections, and is well-qualified to form an opinion on the value of any remedy employed for them.

His present communication is on an extract of the piper cubeba, prepared by Mr. John Toller, in the following manner:—

The cubebs are exhausted by repeated digestion in alcohol, which readily takes up all the active principles of the pepper, viz. a resin resembling that of copaiba, and a coloured resin, with an almost concrete essential volatile oil. The alcohol is distilled off from these tinctures at a temperature so moderate as not to volatilise the essential oil: when the operation can be conducted no further in this manner, the operation must then be carried on in an open vessel by the aid of a water-bath, at a still lower degree of heat; a little finely pulverised Spanish soap should now be added, to prevent the separation of the resin and preserve the extract of an uniform consistence; with careful attention to the temperature, it is thus brought to the requisite consistence for forming pills.

Mr. Judd believes that this extract retains the effective part of the pepper, without its inconveniences. When taken into the stomach in 15 grain doses, it occasions an aromatic warmth in that viscus, a sense of coldness in the fauces, urethra and rectum, similar to that produced by peppermint, a little quickness of pulse, a diuretic effect on the kidneys, a peculiar smell in the urine, and a rapid diminution in the discharge from the urethra. A single dose of five grains, in a healthy person, will, in two hours, impart to the urine a peculiar well-known odour, and that continued doses will impress the urethra with a cooling sensation, and, in a few days, arrest its diseased secretion. In short, it appears to possess all the properties that are known to exist in copaiba, turpentine, and this class of remedies.

Mr. Judd divides gonorrhœa into three stages. The first is seen "about the third day after contamination." A slight redness surrounds its orifice, while the inflammation arrives at that height which produces a thin colourless discharge, with a soreness and irritation in the urethra. The second stage may be known by the increased scalding, the diminished stream of urine, the frequent desire to make water, but above all by the thicker yellow discharge. The third stage is that so well known as "gleet."

Mr. Judd adds :—

"Cubebs will always be found capable of lessening or removing the very first issue of discharge and the irritation accompanying it, whilst the former is thin, and before the inflammation in the urethra has become sufficiently great to induce the formation of puriform fluid. If cubebs be taken but an hour later, they only serve to aggravate the disease in its second stage.

Cubebs again are highly beneficial when the discharge is great in quantity, but thin and transparent; and therefore, in the third stage, they often act as a charm and effect a cure in a few days.

Cubebs have another invaluable quality, that of being applicable and beneficial to the stages of urethritis in which copaiba aggravates all the symptoms; and, lastly, when this remedy has sometimes been found ineffectual in thoroughly removing the discharge, it renders the disease so mild that turpentine and copaiba are then found to be, by it, rendered certain in their curative effect." 32.

Mr. Judd relates six cases which appear to have been treated very successfully by the preparation of cubebs under consideration.

We would observe that, so far as we can judge, cubebs, though a valuable, is an uncertain remedy. It seems to act extremely well on some persons, and to be of little utility to others. Those who have had much experience in the treatment of gonorrhœa must, we think, have remarked, that with some patients cubebs generally answers, with others copaiba, and with others injections. Why this should be we cannot of course pretend to say. We quite agree with Mr. Judd on the *general* inapplicability of cubebs to the inflammatory stage of gonorrhœa. Not that it will always aggravate it, for some surgeons give cubebs through thick and thin, and they occasionally succeed with cubebs or copaiba, given in the stage we have mentioned. But we are sure, that the *balance* of advantage preponderates in favour of abstaining from stimulating remedies in that stage, and, in the long run, the surgeon will effect most good and least mischief who does so.

We are not so certain of the correctness of the assertion, that "cubebs have the invaluable quality of being applicable and beneficial to the stages of urethritis in which copaiba aggravates all the symptoms." We have not observed any thing which would justify us in making so positive an affirmation. Indeed it is indirectly contradicted by a previous remark of Mr. Judd's—*that*, cubebs will aggravate the inflammatory stage. That is the stage which copaiba is apt to aggravate too, and then what stages remain to be adapted for the one medicine and not to be adapted for the other?

P. S. We have tried the extract in a few cases with indifferent success.

### II. On the Use of the Essential Oil of Turpentine in the Treatment of Iritis. By John Foote, Jun. Esq.

Mr. Foote believes that turpentine acts, in these cases, by inducing irritation in the mucous membrane of the intestinal and urinary canals. He quotes and approves of Mr. Carmichael's formula for its administration.

℞. Olei terebinthinæ rectificati ℥j. vitellum unius ovi; tere simul, et adde gradatim emulsionis amygdalarum ℥iv., syrupi corticis aurantii ℥ij., spiritûs lavandulæ compositi ʒiss., olei cinnamomi guttas tres vel quatuor. Misce; sumat cochlearia larga duo ter de die.

In a few cases it has been necessary to increase the quantity of turpentine to an ounce and a half or two ounces in the above mixture, the other ingredients being proportionally diminished, so that a drachm and a half or two drachms of it may be taken each time; but, in general, when administered to the extent directed in this formula, it has very seldom indeed failed, though extensively tried, and in very urgent cases.

Mr. Foote relates five cases, all of which occurred at the Westminster Ophthalmic Hospital. They tell, more or less, in favour of the medicine. Perhaps the case we shall introduce is as strong that way as any.

*Case.*—Wm. Chalfont, ætat. 18, admitted July 21, 1829, with syphilitic iritis of the eye. He reports himself to have had sores on the penis some time since. He has now a copper-coloured eruption (the lichen syphilitica) very plentifully on the back and arms, accompanied with nocturnal pains in his shin bones. He has not had a sore throat, nor has he taken mercury. His eye has been inflamed for three days, and he suffers severe circum-orbital pain, especially at the lower part: vision is also considerably impaired. The iris is much changed in colour, and is immoveable: pupil regular: conjunctiva inflamed: and the vessels of the sclerotic are of a pink colour, and very numerous round the margin of the cornea, forming a beautiful zone, with a white inner circle immediately surrounding the cornea.

℞. Olei terebinthinæ drachmam: capiat æger ter in die.

22nd. Has taken his medicine regularly: makes water more frequently, but without any pain: urine high-coloured. He suffered much pain in the eye last night, from which he is now quite free. The iris is not so much discoloured, but the vascularity of the conjunctiva and sclerotica is just the same as yesterday. Says the medicine makes him feel sick. Continue.

23rd. The circum-orbital pain is as severe as ever, but the inflammation is lessened and vision is improved. He complains of great pain in the glans penis during micturition: urine bloody and turbid. Pergat. Habeat infusum lini ad libitum.

24th. Pain round the eye not diminished: the iris is becoming irregular, and more changed in colour, with small globules of lymph adhering to its surface. He has frequent calls to empty the bladder: urine bloody on voiding it. He suffers extreme pain in the hypogastric region, extending also along the urethra to the glans penis. Let him be cupped to ten ounces from the temple, and continue his medicine.

25th. Would not be cupped, and did not take his medicine yesterday: he sees better, and does not complain of any pain: the vascularity is not so great: the strangury continues severe. Pergat in usu olei terebinthinæ et infusi lini.

28th. Had a purgative on the 26th. Has not taken the turpentine regularly since the last report: he has not any pain in the eye, and vision is quite restored. The urinary organs continue highly irritated: the eruption is paler. Instilletur gutta solutionis belladonnæ. Habeat pulveris jalapæ compositi drachmam.

30th. The strangury is lessened: the eye continues free from pain, but the vascularity is not removed: the iris has nearly regained its colour, but is irregular. Let him re-commence the turpentine.

The terebinthina was used for a short time with decided advantage: he again omitted it too soon, and was obliged to resume it. When discharged, the redness had disappeared, the iris had regained its natural colour, and the pupil was regular.

We conceive there can be no doubt that, in ordinary cases of iritis, calomel and opium are the best remedies. But in certain instances their use may be precluded by constitutional peculiarity, or by cachexia, and the knowledge of another remedy, inferior though it may be, is useful. Turpentine appears to be such a medicine. Not comparable, under common circumstances, with mercury, it is still possessed of some degree of efficacy, and may at times be given with advantage.

### III. Sir Robert Kerr Porter on the Cuichunchulli Plant, and its Effects in Elephantiasis.

Sir Robert communicates the following account of the cuichunchulli, or *viola parviflora* of Mutis, transmitted to him by a physician of Venezuela. It has acquired the highest popular reputation in the treatment of elephantiasis, and eruptive disorders of that class.

Its medicinal qualities, like those of the *viola ipecacuanha*, reside in the root. It may be pulverized and given in doses of half a drachm, mixed with a little warm water: this dose is administered during the first days, or until the system is seen to become sensible to its effects. In order to encourage the perspiration it excites, the patient ought to take several cups of the infusion of the alder, another sudorific plant. Frequently it produces nausea, and even vomiting, lowness of spirits and great anxiety of mind; violent perspiration, and copious discharges of urine, as well as a strong salivation.

It is requisite that the patient be kept close to his room during the time of administering this remedy.

The number of doses, as well as quantity, must be augmented according to the effects produced; its use ought to be prolonged for a period as symptoms and circumstances appear to call for.

"As yet," says the cautious physician who writes to Sir Robert Porter, "I have not seen any decided cases of elephantiasis cured by administering this plant. In two cases I certainly perceived much alleviation. In one of them was observable an almost total disappearance of the greater part of the symptoms of leprosy, which had already far advanced; but it was not either a complete or satisfactory cure. In the other I remarked some favourable change on the spots of the skin, on the ulcers of the nose, and in the pimples. Jointly with this remedy was used washing the interior of the nostrils with a solution of subborate of soda and corrosive sublimate.

I have tried the cuichunchulli, and seen it tried in four or five cases, but, with the exception of the two I have stated, (where I was called in at a consultation with other medical men), its effects were null, or almost imperceptible.

Many other medicinal virtues are attributed to this plant, but without denying all of them, I must venture to doubt them, or at least to believe, they are greatly exaggerated."

We prefer this plain, practical, and philosophical statement to the florid accounts of wonders effected by the cuichunchulli, contained in other documents forwarded by Sir Robert. At the same time, the medicine having very obvious purgative and emetic effects, is evidently not an inert one, and may turn out to be an useful one.

We shall only add a statement of Dr. Farr's, that:—There is now in this country a considerable quantity of the true cuichunchulli besides the specimens presented to the Society. Mr. Blundell, of Liverpool, has received from Guayaquil a parcel of one hundred and forty pounds weight, collected about Riobamba. Sir R. Porter states that a small quantity of the root was sold at Corveas for the enormous sum of £3 6s. per ounce, i. e. for nearly its weight in gold. Mr. Blundell, is of opinion that it cannot be sold in this country for less than 10s. per pound, or about one hundredth of that price; yet he infers, from even this cost, that it is a scarce plant, and the expense of collecting the root considerable; and, in fact, the Venezuela Gazette states, that it is not produced abundantly. Those practitioners who like to try it have the opportunity.

A PRACTICAL TREATISE ON THE HUMAN TEETH, SHOWING THE CAUSES OF THEIR DESTRUCTION AND THE MEANS OF THEIR PRESERVATION. By *William Robertson.* London, 1849, pp. 205. Second edition.

THE work before us, already well known to many of our readers, is materially different from the current brochures on the management of the teeth, and lays claim to the first promulgation and to the first practical application of a theory of caries or decay of the teeth, which is likely materially to stamp and improve the practice of dental surgery. It is well known that Bell and Fox, the chief modern writers of consideration on the teeth, attribute the loss of these organs by decay, to inflammation, seated either in the lining membrane, or proper bone of the tooth. Mr. Robertson disagrees with this, and by a number of proofs drawn from the structure of the teeth, their physiology, development and the operations performed upon them, establishes, that caries is the result of the chemical action of decomposed food upon the teeth, and not the result of inflammatory action, which had been previously supposed.

"Upon examination it will be found that there are fissures formed in the enamel of the teeth, in consequence of the irregular distribution of that substance upon the surfaces; and that there are also interstices produced by the crowded position of the teeth, and irregularity of their shape. In these situations the particles of food are retained, which undergo a process of decomposition, and acquire the property of corroding, disuniting, and thereby destroying the earthy and animal substance of which the teeth are composed. That this is the cause of the destruction of the teeth, commonly called 'caries,' and that it is not the result of inflammation, either in the membrane or the bone of the tooth, will appear obvious from facts which will be produced, and which I have daily witnessed during a long course of practice."

The facts here alluded to are chiefly to be sought for in the formation and individual structure of particular teeth, hence we find that the more irregular is the surface of a tooth, and the deeper the fissures in the enamel, the more likely is it to be the subject of caries, thus the dentes sapientiæ are the first generally to decay, next the molares, then the bicuspides, till we find that the incisors, particularly those of the lower jaw, are hardly liable to caries, except under very particular formations, depending upon a deficient deposition of enamel on their cutting or grinding surfaces.

"It is to this irregularity of structure, so peculiar to the double teeth, that their tendency to caries is to be attributed; and the liability of the teeth in different individuals to decay will be in proportion to the form and depth of these fissures. On the other hand, where there is a close union of the section of enamel upon the surfaces of the teeth, there will be no tendency to decay, because no lodgment of food can take place."

Mr. Robertson next proceeds to point out the situations upon the teeth, in which decay most commonly takes place, and to explain the cause why one class of these organs is more liable to decay than another. The teeth are then reviewed in the order of their liability to decay, which is, as we have said, first the molares, then the bicuspides of the upper jaw, afterwards the incisors of the upper jaw, and lastly, the least disposed of all are the incisors of the lower jaw. The reasons why this is the case are fully explained by Mr. R. in an elaborate account of the formation of the teeth, and the disposition of the enamel upon their surface. This is ably done, and the proofs, drawn from the anatomical structure of each class of teeth, appear to us so conclusive, that we are disposed to believe them incontrovertible.

"From the review," says our author, "we have just taken of decay in the different classes of the teeth, it will be perceived that, in regard to situation, it takes place on the surfaces of the teeth, in excavations formed between them and the projecting gum, in cavities, indentation and irregularities, on the external surface of the tooth itself, and that it occurs at their sides, in their necks, and spaces produced by their formation and relative position ; in regard to frequency, that it is in proportion to the depth of the superficial depression, and the degree and nature of the lateral projections and interstices.

This being the case, and decay never being found to take place upon the plain and smooth surface of the tooth, it cannot for a moment be doubted, that the predisposition to caries depends upon the external configuration or conformation of the teeth. It must be equally evident from the partial nature of the disease and from the insufficiency of all general causes to explain this circumstance, that the exciting cause of caries must be one whose operation is partial, and which has a peculiar action upon those parts of the teeth, which are by their structure predisposed to decay. The only cause capable of explaining the partial operation and the particular situations of decay, is the corrosive or chemical action of the solid particles of food which have been retained, and undergone a process of putrefaction or fermentation in the different elopes of the teeth best adapted for their reception."

Following out the theory now stated, Mr. Robertson shews why teeth are so liable, and so frequently decay in pairs, from opposite sides of the jaw ; why also, decay is more frequent in youth than in age, and why there is a great susceptibility to decay in the teeth of particular families. All these circumstances arise from the original anatomical disposition of the surfaces, and situation of the teeth, from the similarity of the teeth on opposite sides of the jaw to each other, and from the simple fact that the deeper the fissures, the greater quantity is the food retained, the more quickly is it decomposed, and the tooth decayed and lost. The various proofs in support of these several positions are detailed in the fifth chapter of the work, and are well worthy the most attentive perusal.

The description of the mode in which decay commences, and proceeds till the external cavity of the tooth is perforated, tooth-ache produced, and the organ inevitably lost, we consider of very great importance, and shall let the author describe it in his own words.

"When decay commences upon the surface of a grinding tooth the corrosive action is confined to one or more of its deepest indentations, and may be discovered by a brown discolouration of the part, which is produced by a chemical action of the food lodging in this situation, and as this action proceeds and increases, the bottom of the indentation becomes black and corroded, and soon afterwards a small opening may be discovered through the enamel. When the putrid substance has been admitted through this orifice to the softer bone within the enamel, decomposition in this situation proceeds with much greater rapidity, whereas the opening through the enamel increases but little, its texture being better able to resist the effects of chemical action, on account of its greater density ; but when decay has proceeded so far as to make an excavation, by the destruction of a considerable portion of the bone under the enamel, the support being removed, the enamel suddenly breaks in during mastication, and a cavity is laid open, which till this period was not suspected, in consequence of the smallness of the orifice, and the destruction of the tooth, up to this stage of decay, being unaccompanied with pain. At this period or soon after, by the continued operation of the same chemical action, the internal membrane that lines the cavity of the tooth becomes exposed, and consequently inflammation occurs, the pain produced by which is tooth-ache."

Through the 7th, 8th, and 9th chapters of the first part of the work which are devoted to the explanation and support of the theory insisted on, Mr. Robertson proceeds to deduce further proofs in support of its validity and soundness. These are chiefly drawn from the nature of the operations performed upon the teeth, from a comparison of the human teeth with those of some animals, and from some further considerations on the inorganic character of the bone of the tooth.

The second part of the work before us considers the application of the theory to practice, and here Mr. Robertson insists upon the frequent inspection of the teeth by a dentist, for the purpose of preventing decay proceeding so far as the destruction of the bone of the tooth, and the perforation of its cavity, at which period the case is hopeless, and the tooth must be inevitably lost,—at all periods previous to this art can preserve it.

"I have before stated," says Mr. R. "that between the ages of six and twelve years the teeth require most attention; because, during this period it is necessary to attend to their arrangement, as well as to detect and arrest caries in time; and moreover, during this early stage of life, there is the greatest tendency in the teeth to decay. There are some persons whose teeth are altogether exempt from caries, and require no assistance from the dentist. But it will be found in cases of this kind that the teeth are regular in their arrangement, and that the sections of the enamel upon their surfaces are closely united; therefore they do not present receptacles for the food to lodge in, and, consequently, there is no tendency in such teeth to decay."

The act of preserving the teeth is to keep them clean, and, if this can be accomplished there will be no tendency in them to decay. It is not however the mere surfaces that must be kept clean, but the natural pits and fissures, which the natural arrangement or construction of the teeth form.

"It would be well if persons would look into their mouths, and make themselves familiar with the forms and arrangement of their teeth, in order that they may ascertain the practicability or impracticability of keeping them clean by means of the tooth-brush. At all events, a knowledge of the structure and position of the teeth would enable them to apply the brush in a way best calculated to effect this purpose; for there is, even in the right performance of this simple operation, some little art required. The attention should be particularly directed to the grinding surfaces of the double teeth; these are the most subject to decay, because of the irregularity of their structure, and consequently their tendency to retain food."

We recommend the careful perusal of the above treatise which is strictly practical, and even popular in its character to all persons interested (and who are not) in the preservation of their own teeth or that of their children. We firmly believe that a general acquaintance with the principles of Mr. Robertson's theory, and the consequent periodical examination of the teeth, which such an acquaintance would produce, must tend most materially to diminish the frequency of a most painful and annoying disease, and render the economy of the mouth an object of daily and profitable attention. The simple and clear manner in which the facts contained in Mr. Robertson's book are set forth, their important application, and the facility, with which the proofs brought forward in support of the theory, may be comprehended by even unprofessional readers, must render the present publication not only an important improvement in modern dental surgery, but also a valuable and useful contribution to the public at large.

Illustrations of Cutaneous Disease.—A Series of Delineations of the Affections of the Skin in their more interesting and frequent Forms; with a practical Summary of their Symptoms, Diagnosis, and Treatment, including Appropriate Formulæ. By *Robert Willis*, M.D. Licentiate of the Royal College of Physicians, Physician to the Royal Infirmary for Children, Author of an English Version of Rayer on the Diseases of the Skin, &c. Fasciculi II. to XV. Balliere. London, 1839.

We noticed the first Fasciculus of this valuable series of Illustrations, on its appearance, and take shame to ourselves for having omitted to bring the subsequent ones before our readers. But we shall make both apology and atonement at the same moment and by the same act.

Fasciculus II. presents us with plates of *Lepra Vulgaris*—of *Pemphigus*—of *Purpura*, in the shape of, 1, *Ecchymoses*, and 2, *Petechiæ*.

We shall allude to that portion of the text which discusses, or, rather, hints at the treatment of Lepra Vulgaris. We do so because that treatment is difficult and far too frequently unsuccessful. Dr. Willis, we think, overrates its curability very materially, when he affirms that perhaps in the majority of cases the disease may be wholly eradicated. In the young it is undoubtedly more curable than when the season of youth is past; yet, even in the young, it is but too prone to relapse.

Dr. Willis observes that it is well to begin by abstracting blood; first in proportion to the age and strength of the subject, and next to the amount of pain and irritation endured from the disease. This is to be followed up by the exhibition of adequate doses of purgative medicine, (and there is nothing better than a few grains of calomel with some compound extract of colocynth at night, followed by the common black draught of salts and senna next morning,) at intervals for ten days or a fortnight afterwards, the patient being meanwhile kept upon bland farinaceous and unstimulating food, and enjoined the use of some mild diluent with liquor potassæ or carbonate of soda. These measures continued for a few weeks have often a very marked influence on the disease; and intermitted for short intervals, and then resumed, are sometimes of themselves adequate to subdue it entirely. It is quite consistent with our observation to place the main dependence on remedies of this class—evacuants and such as improve the secretions. But they relieve rather than cure.

After a time, it becomes proper to try some of the more empirical medicines—the tincture of cantharides—the liquor arsenicalis, the general favourite. Dr. Willis says, that "the farrago of vegetable infusions and decoctions that have been recommended in Lepra, do not appear to have any influence beyond that which they possess as simple diluents." We can by no means concur with him in ranking sarsaparilla as a simple diluent, nor are we sure that the dulcamara, or the taraxacum, can be considered so. No doubt they are each and all prescribed with very vague, very empirical, and very exaggerated notions; but they have an action, the first and the last at all events, notwithstanding. The sarsaparilla is often beneficial after persons have been a good deal lowered, and in those of lax fibre, or of weakened health, it is of service. But quoad the lepra it must be cautiously used, the vascular fulness it tends to produce being hazardous.

Dr. Willis says little, yet not too little, in favour of local applications. The general warm bath, he remarks, is useful in removing squamæ and softening the skin. The patches cleared by its means sometimes improve obviously by being rubbed twice a day with a salve of white precipitate, or with an unguent of iodide of sulphur; or by being moistened with strong acetic acid. They are perhaps even more advantageously treated by having a rod of the solid nitrate of silver passed rapidly over the healthy integument round them, the parts having been previously moistened. And he concludes by stating that the vapour and sulphur bath deserve a trial.

So far as we have seen, no good is done without warm baths. The skin is, in most instances, dry, and baths tend to restore its functions. But we never *saw* any good from the sulphur bath. *That* tends to make the skin more dry, if administered effectually; and if ineffectually, it is, of course, a delusion.

The Third Fasciculus represents *Eczema Capillitii, Pityriasis Capitis, Ecthyma Capillitii,* and *Porrigo Scutulata* (of Willan), or ringworm of the scalp.

We may say a word or two on eczema of the scalp, a complaint very frequently misunderstood and mismanaged. As Dr. Willis remarks, the elementary, vesicular form of the eruption, and the discharge of serous fluid, distinguish eczema from any other affection that has its seat on the scalp. Even when the disease has passed into the chronic and furfuraceous state, its history in previous stages, the tender and glistening appearance of the integument, and the nature of the scales—partly cuticular, partly dried exudation, are characteristic of the case.

Dr. Willis counsels, the complaint being left to run its course. This must not, however, be understood literally, for to prescribe alteratives and aperients is to interfere as much, as to apply escharotic washes. The *mode* of interference differs in the two cases; but interference there is in each. Dr. Willis remarks that he has never seen repellent means adopted in an early stage of the complaint without more serious symptoms following. It is better to prescribe some mild alterative every night or every other night—a few grains of rhubarb and carbonate of soda—and the occasional use of a mild diaphoretic mixture in the course of the day, containing the acetate of ammonia or citrate of potash combined with the tartrate of antimony in small doses—and to await the natural decline of the eruption. The only topical medication admissible consists in tepid sponging, and great attention to cleanliness. Incrustations are best got rid of by the application of a bread and water poultice, and subsequent bathing with warm water. When the disease declines, a weak solution of sulphate of zinc (two or three grains to the ounce of water) becomes a useful tonic application. And when it has passed into the stage of repeated flimsy desquamation, a salve of the white precipitate (gr. v.—x.—xv. to the ounce of lard) is always of signal service. It has appeared to us that in all cases of eczema, and we might even extend the proposition to most diseases characterised by a tendency to serous exudation, evacuating medicines are necessary. Rather smart purging and diuretics have always seemed to us to be of service. The liquor potassæ is a valuable medicine in these cases, and chiefly, we think, valuable when it acts upon the kidneys. So soon as the system has been freely unloaded, lead in the shape of lotion, poultice, or ointment, agrees as well as any local application, if not better than most. Irritating applications do not answer so well.

Infants often suffer from the disorder during the whole time of teething. Yet it should not be allowed to pursue its course unmolested on that account; it may degenerate into a very obstinate affection. Regulation of the secretions, great cleanliness, and tepid poultices, are requisite.

*Porrigo Scutulata*, or trichosis simplex, or ring-worm, has, for its essence, inflammation of the hair-follicles. Dr. Willis observes, very justly, that the disease is therefore erroneously, although almost universally, described as commencing with the formation of the pustules denominated achores and favi. When it ends in suppuration, the pustules are of the description mentioned; but they are still accidental, not necessary to the disease. He has often not seen a single pustule from the beginning of a case to the end of it.

As to treatment, our author confesses his ignorance of a specific. The disease goes on for a certain time, in spite of every thing that can be done, and yields at last to what it may have resisted at first. In the earlier stages, Dr. W. advises tepid fomentation for an hour or more at a time, and repeated once, twice, or thrice in the course of the day, and the application of a light bread and water poultice during the night. The complaint will occasionally yield to this alone. The other remedial hints which our author gives are the following:—The lunar caustic applied in a ring round the patches at the distance of about a line from their outer margin, has also occasionally a very remarkable effect in modifying the morbid action that is going on within them. Applied directly to the parts affected, however, this and all other irritating or stimulating washes and salves do mischief in the earlier periods of the disease. By and by they become as useful as in the first instance they were prejudicial. A solution of sulphate of copper (gr. vi.—x. to the ounce of water), of nitrate of silver, (in the same proportions,) the mild ointment of the nitrate of mercury, a salve of the black sulphuret of the same metal (sulphuret. hydrarg. nigr. ʒj.—ʒij. adepis ℥j.) the unguentum picis, an unguent of the cocculus indicus, (coccul. ind. pulveriz. ʒj.—ʒij. adepis ℥j.) may be tried one after another, and in different instances, each will have the credit of the cure. Time and perseverance are the sovereign remedies, combined with which every other succeeds, without which all in their turn will certainly fail. The most efficacious remedy in itself is undoubtedly the eradication of the affected hairs; these are to be removed singly with forceps. We have seen as much benefit from the ointment of the iodide of sulphur, as from any thing. It should be rubbed upon the part some two or three times a day, and conjoined with the frequent use of warm soap and water.

When the part affected is not particularly covered with hair, the application of a solution of sulphate of copper or nitrate of silver is generally effectual.

Fasciculus IV. depicts *Psoriasis Discreta s. Guttata—Herpes Phlyctænodes—Scabies Purulenta*—and *Strophulus Confertus.*

Dr. Willis says of the treatment of Scabies:—the partial and scanty employment of the sulphur ointment are the errors to be avoided; it should be used in large quantity and generally. The patient should stand before a good fire and anoint the whole surface of his body, and especially those districts that are more peculiarly affected, with abundance of sulphur ointment (equal parts of sublimed sulphur and lard). He should then dress himself, without washing off any of the ointment, in a complete suit, consisting of drawers, a calico shirt, stockings and gloves, and go to bed. There he should remain for twenty-four hours, after which he may rise and step into a warm bath, where he is to cleanse himself by means of white soap from all traces of the grease. He now dresses in clean garments, and in the vast majority of instances he will be no further troubled with his disease, unless he contracts it again by putting on any of the infected raiment he may have formerly worn.

Common as scabies is, we have been surprised at the frequency with which it is mistaken. We are every now and then consulted by patients, who have been seen, perhaps, by several medical men, without *their* discovering the nature of the case—the itch. We cannot say, however, that we have found the complaint so curable as Dr. Willis would imply. Our patients have required more than one night's brimstone, and we suspect the majority will not be let off so cheap. Indeed we have found the simple sulphur ointment much less effectual than the compound. The latter produces a good deal of irritation, it is true, but this disadvantage is counterbalanced by its curing the complaint.

After enumerating the remedies that are recommended for *Psoriasis discreta,*

remedies almost identical with those employed for lepra, Dr. Willis believes that arsenic is of most efficacy in psoriasis. Admitting the tendency to relapse, he adds:—"The only efficient mode of guaranteeing the patient against this, is to conclude the cure by the administration of some eight or ten sulphur-fume baths, which, as the most powerful of all the means we possess of influencing the skin, deserve to be much more freely administered than we have either the opportunity, or, it would seem, the inclination to do in this country." We would take the liberty of repeating the remark we have already made, that, so far as we have seen, the sulphur fumigation has a tendency to render the skin dry, and is not likely to be productive of much benefit in cases of scaly eruption, attended, as they usually are, with such a condition of the surface. Discrimination should, therefore, be exercised in the recommendation of this remedy.

FASCICULUS V. portrays *Impetigo Capillitii—Trichosis Favosa—Trichosis Lupinosa—Trichosis Decalvans.*

Of Impetigo Capillitii, Dr. Willis says, that it presents itself with the characters appropriate to each of the forms that have been described by nosologists, viz. in single insulated pustules—Impetigo sparsa, in oval or circular clusters of pustules—Impetigo figurata—and in rings, Impetigo annulata. The Porrigos and Tineas of authors are in the majority of instances referable to Impetigo in one or other of its forms. Impetigo figurata he has principally seen in children about or under a year old; the distinct is the more frequent form, (Impetigo sparsa,) and occurs later in life—from about the fourth to the tenth year. In none of its forms is the disease contagious. It is allied in character and in treatment to eczema, into which it runs.

The Trichoses, already noticed in part, are a very obstinate and therefore an interesting class of eruptions. The trichosis favosa and trichosis lupinosa correspond to the porrigo favosa and porrigo lupinosa of Willan.

"Neither the trichosis favosa," says Dr. Willis, "nor its derivative the trichosis lupinosa, are very common diseases in this country. Several months will often elapse before a single case of the trichosis (porrigo) lupinosa is observed among the thousands of out and in-patients who present themselves at the different hospitals of the metropolis in the course of every week. In France, however, M. Rayer informs us, that next to eczema and impetigo, favus is the most frequent of all the affections of the scalp. Pustular trichosis, however, is by no means confined to the hairy scalp. The follicles of the beard are often affected precisely as those of the scalp are in the disease which I have described as the trichosis favosa, a case in which the affection has hitherto been confounded with the proper sycosis menti. The trichosis (porrigo) lupinosa is well known to show itself occasionally upon the cheeks, neck, bosom, and extremities.

These pustular forms of trichosis, as well as the trichosis simplex, are contagious diseases. They are frequently to be traced from individual to individual, and seem to have the power of engendering one another. I have, for instance, seen the trichosis lupinosa arise on the shoulder and arm of a girl fifteen or sixteen years of age, who was in the habit of fondling and nursing her sister, a child of seven or eight, affected with trichosis simplex of the scalp."

In the treatment of these affections, our author advises mercurial purgatives, followed by light bitters with alkalies. "The hair," he adds, in reference to local treatment, "should be removed from the whole scalp by means of flat scissors—the excitement of shaving does mischief, and should be avoided. Inflamed parts must be soothed by tepid sponging, scabs and incrustations of every kind got rid of by poulticing, and dead hairs, wherever they appear, extracted; pustules should, at the same time, be sedulously and closely looked for once or twice in the course of each day, the hairs, with the roots of which they seem connected, pulled out with a pair of fine forceps, and favous deposites squeezed from their conduits. Should any difficulty be experienced in softening and getting rid of the scabs in consequence of their thickness and hardness, a few folds of linen dipped in a wash of the subcarbonate of potash, (two or three drams of the salt to a pint of water,) or of the hydrochloric acid, (acid. hydrochlor. dilut. ℥j.—℥ij. aq. Oj.,) applied for six or eight hours, will be found effectual in softening them. The tender parts, after they have been bathed with tepid water for some time, may be covered with pledgets of linen spread with the zinc ointment. By and by they may be washed with a weak solution of the sulphate of zinc in water, (four, six, or eight grains of the salt to the ounce of water,) or of the nitrate of silver in the same or somewhat smaller proportion, which will be found very effectual in allaying morbid action and in giving tone to the tissues affected. These means alone, indeed, diligently enforced, are adequate to subdue all those forms of scalp affection in which the hair-follicles are implicated. It is even doubtful whether the cure is materially expedited by the use of stimulating ointments of any kind. It is only in cases which prove indolent, which continue under treatment for more than three or four weeks, that I believe it necessary to try some one or other of the infinite number of unguents that have been recommended in the cure of scald head."

Dr. Willis alludes to the opinion, a disposition to entertain which appears to have prevailed of late, that the trichosis decalvans is a mere consequence of other antecedent affections of the scalp, especially of the trichosis simplex. He believes, and we think with perfect truth, that the hair-bulbs in certain instances are liable to be affected by a kind of paralysis of their functions, in which they shrink and become anemic and atrophied without antecedent inflammation or any one of its consequences. In this event the disease, the trichosis decalvans, shows itself primarily as a spot of variable extent from which the hair has fallen off more or less suddenly, and around the edges of which it continues to be detached for some time after the affection is particularly noticed, the skin of the patch being all the while preternaturally tense, smooth and white, and appearing sunk below rather than raised above the general level of the integument of the scalp. The essence of the disease seems to consist in a simple suspension of the functions of the hair-follicles. The follicles themselves are not destroyed.

This, like other forms of trichosis, appears to be contagious. It is well, therefore, to remove the hair from the whole scalp either by clipping or shaving, and to keep the surface perfectly clean by the use of white soap and water night and morning. By and by the bald patches may be attacked more particularly; though they will usually be found at first to resist every application that can be thought of. Almost the only article Dr. W. has seen of service in the earlier stages is the unguentum hydrargyri camphoratum rubbed lightly with the point of the finger over the parts affected every night. Subsequently, more stimulating applications become useful; a mixture of the soap-liniment, oil of turpentine, and tincture of cantharides, (linim. sapon. ℥j. ol. terebinth. tinct. lyttæ āā ʒij.) often answers extremely well in arousing the dormant energies of the hair follicles. We have seen the iodide of sulphur ointment beneficial. The cold shower bath is useful too. But it must be confessed that the complaint is a troublesome one.

Fasciculus VI. contains *Psoriasis Annularis—Roseola Annulata—Impetigo Figurata* (any thing but happy)—and *Lupus Exedens*.

In a case of *Psoriasis Annularis*, the following treatment has proved serviceable. Dr. W. recommended the patient, who had laboured under the complaint for some years, to lose ten ounces of blood at once; to take a couple of pills containing four grains of calomel, and ten grains of the compound extract of colocynth, at night, and next morning a saline powder consisting of two drams of soda tartarizata and the like quantity of bitartrate of potash with one-twelfth of a grain of tartrate of antimony, twice in the course of each week; to restrict herself to a milk and farinaceous diet, and to anoint the more irritable and itchy patches with the ointment of the ammonio-chloride of mercury at bed-time.

The improvement within ten days was considerable, and the plan was persevered in for ten days more. The effect of fumigating the arms with a mixture of sulphur and iodine was then tried. This proved most beneficial. The eruption on the arms to which the fume douche had been applied, was nearly gone by the end of a week; so that he was encouraged to try the general sulphur fumigation bath. The patient has used these baths six times with much advantage.

In a child about eight years old, who had laboured under epileptiform fits since the age of two years, and under *Impetigo Figurata* for three months, "I directed," says our author, "simple tepid bathing of the parts affected night and morning for a week, and a course of the decoction of the burdock-root, (two ounces of the root to be slowly boiled in a pint and a half of water till reduced to a pint,) two ounces to be taken night and morning. By and by the patches of the disease appeared less irritable; there was no fresh eruption of pustules, and I ventured on the application of a greatly diluted ointment of the nitrate of mercury. This seemed to agree, and the treatment steadily pursued was finally followed by success: the skin began to clear at the end of a fortnight, and before the lapse of a month the only traces of the eruption that remained were a few dusky-red blotches."

We wish we could say that Dr. Willis gives us any hint on the treatment of that disgrace to surgery, *Lupus Exedens*. We shall content ourselves with enumerating the caustic applications that he mentions:—the nitric acid, the acid-nitrate of mercury, the nitrate of silver, the arsenical paste, (arsenic acid 10 grains, red sulphuret of mercury ʒij. charcoal 5 grains,) the compound powder of calomel and arsenic acid, (calomel 99 parts, arsenic acid 1 part,) the chloride of zinc, (chloride of zinc 1 part, plaster of Paris 2 parts,) are those that have greatest efficacy, and are most commonly employed. A formidable catalogue of unsuccessful escharotics.

FASCICULUS VII. contains *Psoriasis Inveterata*—*Vitiligo*—*Purpura Urticans* and *Lichen*.

In the case of supposed *Vitiligo*, from which the present drawing has been taken, a case which was under the care of Sir Benjamin Brodie, in St. George's Hospital, the patches spread irregularly outwards on every side from the spot first affected, presenting a corymbose appearance, as if made up of many smaller rounded patches, and at their height having a pretty bright purple hue. The patches were slightly raised above the general level of the skin; they were not painful; but not insensible; the patient knew when they were touched, and, at times, they were somewhat itchy. The patient was affected with varicose veins of the affected leg; and on different occasions the vitiliginous patch give way, and, in parts, become an open sore, discharging a sanguinolent serum; the sores, however, were never of bad character, for with rest and simple dressings, the wounds always healed readily. A variety of applications were tried to the affected part; that which seemed to do most good was naptha; under the use of this the principal patch sank nearly to the level of the surrounding skin, and the colour faded to a tawny or pale purplish brown. The general health of the patient appearing to suffer from his residence in the atmosphere of an hospital, he was discharged improved, but not entirely relieved of his disease. It may not be unimportant to observe, that the disease first showed itself in the United States of America, a country in which the patient had resided for several years.

In reference to the treatment of *Lichen*, Dr. Willis says:—In the milder cases, rest, a cooling regimen, the avoidance of all causes of excitement, such as a hot atmosphere, a warm bed, &c.; and the continued use of some mild diluent, such as lemonade, with the occasional exhibition of a gentle saline aperient, suffice to bring the disease to an end. Should the symptoms run higher, it becomes necessary to detract blood, to purge more freely, and at the same time to prescribe a sherbet of one of the mineral acids. All topical applications should be of the mildest description, and used cold. Sometimes no wash but simple water, a decoction of bran, or a weak solution of gelatin, at the temperature of about 60° Fahrenheit, can be borne. By and by a weak unguent of the white precipitate of mercury may be tried, or what often answers better, one of the black sulphuret of the same metal (ʒss. ʒj. ʒij. to the ounce of lard). The iodide of mercury has also been recommended, and the pruritus may sometimes be effectually allayed by cauterizing the papulæ superficially with the nitrate of silver.

Fasciculus VIII. has *Icthyosis Simplex—Acne Pustulosa—Sycosis—Erythema Fugax.*

Dr. Willis remarks that, several diseases affecting the integument of the face have been described under the common names of acne, mentagra, and sycosis. These diseases have their seats in different elements of the skin. Some of them affect the sebaceous follicles, and belong to the proper Acnes. Others inhere in the hair-bulbs, and are from first to last genuine Trichoses. A third set implicate first the more superficial and then the deeper layers of the dermis, and are accompanied by the growth of soft, fungoid, fleshy masses, moist or crusted on the surface, of a bright red colour, and varying in size from that of a hempseed to that of a cherrystone or hazel-nut. These growths were compared to figs, whence the name of the disease they characterize, Sycosis. In this last disease the sebaceous follicles and hair-bulbs are often altogether unaffected; when they are implicated, they seem to be so secondarily, and in consequence of becoming involved in the inflammation that surrounds them.

Having described the formation of the pustules, Dr. Willis goes on to observe:—

"When from the intensity of the pruritus the parts affected are violently rubbed, pustules hardly make their appearance at all; but in their stead there is an oozing of a pale viscid lymph around the shafts of the hairs, which dries into crusts precisely as in the former case. Little or nothing save a small drop of lymph can at any time be squeezed from the pustules or oozing points, but the hairs which traverse them will in many cases be found either wholly or partially loosened, so as to be easily extracted; and where this does not happen in the first instance, it very commonly occurs at a later period. I know by repeated experience, indeed, that if the disease be narrowly watched, and the hair whose root is affected be singled out and extracted, the malady will cease in so far as this particular hair is concerned. The same remark applies to those cases in which the orifices of the sebaceous follicles are affected: their contents being squeezed out as soon as they begin to prove sources of irritation, the morbid process goes no further. When no attention is paid to the disease, however, or when the surfaces affected continue to be irritated by the application of soap and the daily use of the razor, or by improper stimulating unguents, &c. one crop of pustules succeeds another; the morbid actions extend from those parts in which they were first set up, the entire thickness of the corion, and even the subjacent cellular tissue, become affected, the surface is rendered nodulated and uneven, and under an accumulated mass of crusts and filth, the fig-like, spongy, and moist excrescences of sycosis show themselves, and cause the disgusting appearance and disfigurement that are generally felt as not the least painful part of the disease."

The hint already given in regard to the connexion of the hairs and hair-bulbs, and the sebaceous follicles and their ducts, with many cases of pustular

Mentagra, may often be used to excellent purpose in the treatment of the disease. With few means beyond tepid bathing, and the extraction of loose and dead hairs, I have seen more than one case of Mentagra yield, which had long resisted constitutional measures of every kind. With the exception of extracting hairs whose roots are diseased, the local treatment of this disease in all its stages ought to be soothing—tepid bathing, fomentations with decoction of bran or poppy heads, the constant application of a few folds of soft linen wrung out of one of these decoctions, or of soft bread and water poultices, are the proper means. The beard is at the same time to be kept short by the scissors,—on no account is it to be touched with a razor. The same general principles are available where the sebaceous follicles appear to be the elements principally implicated; and nothing can be more satisfactory than their influence in those cases that are accompanied with the growth of moist ficoid excrescences."

On the constitutional treatment Dr. W. says nothing that we need advert to. We must confess that, occasionally, acne proves a highly troublesome complaint, whatever the method of treatment resorted to. We quite agree with Dr. Willis that, too frequently, these cutaneous disorders are any thing but judiciously managed, and the mistake commonly lies in the over fondness for irritating applications.

Fasciculus IX. presents *Erythema Papulatum et Tuberculatum—Scabies Impetiginosa—Favus Corporis—Urticaria Vulgaris.*

In his observations on the plate of Scabies Impetiginosa, Dr. Willis admits the connection between the various groups of cutaneous disease, vesicles running into pustules, tubercles becoming scaly, &c. Indeed it is this tendency that both multiplies the names of cutaneous complaints, and renders them and the descriptions to match them, always behind the varieties of nature after all. We can hardly help agreeing with those, who think both names and distinctions multiplied too much already. Too numerous to permit simplicity, they never can be numerous enough to form a perfect picture of nature. The broad features of these, as of some other disorders are the only ones that need be cared for at the bed side.

But to return to Scabies Impetiginosa, Dr. Willis observes:—"the patient, a girl only twelve or thirteen years of age, presented herself to me among the crowd at the Infirmary for Children, and I did not doubt from a hasty glance that I had to do with a case of Impetigo, which I characterized as I. Scabida, from the peculiar appearance of the crusts. But the constitutional and local treatment recommended did little good; the disease went on unaltered in its characters. After a time, a more careful scrutiny led to the detection of numerous extremely minute vesicles, and it was now evident that though impetiginous in its more striking features, it was only secondarily so. The routine practice for Scabies—the inunction of strong sulphur ointment—was now prescribed; but in four days the girl presented herself decidedly worse, the extremities so stiff and painful that she could scarcely move them, and she had only walked to see me with extreme difficulty. The sulphur ointment was not repeated; a couple of grains of calomel on alternate nights, followed next morning by a dose of infusion of senna and a neutral salt, and the tepid bath every day, were ordered for a week. At the end of this time the disease was much less irritable, but fresh vesicles and pustules were still appearing, and the pruritus was perhaps more violent than it had ever been. The sulphur ointment diluted, was again adventured on in the manner already recommended, (vide Scabies Purulenta,) and this time with success; within a week there were no more than red traces of the disease to be discovered on the whole of the arms and trunk. On the thighs the disease was not subdued, and there it required a recurrence to the antiphlogistic, constitutional, and soothing topical plan before it could be got rid of, which it was however very completely in the end."

Fasciculus X. depicts *Eczema Acutum—Strophulus Eczematodes—Cancrum Oris*—and *Herpes Iris.*

In a case of acute eczema, which our author witnessed, the eruption extended from the roots of the hair over the forehead and face, including the ears, the whole of the throat, nearly to the line indicated by the outer edge of the trapezius muscle on either side, and the entire anterior aspect of the thorax from the shoulders to the epigastrium. The whole of this extensive surface in a large and rather corpulent man was of the deepest crimson, the surface being everywhere partially concealed by rounded and angular pale yellow coloured, flimsy incrustations, which were here and there moist and pliant, in other places dry and unyielding.

Eczema is so common a complaint, at least in private practice, that accurate ideas should be entertained on the subject of its treatment. Acute eczema it is true, is rare, but *it* occasionally happens, and chronic eczema is frequent enough. Dr. Willis says that, for acute eczema, blood-letting may often be practised with great advantage; although, as the part implicated is not immediately necessary to the actions of life, this measure should never be carried to any length. Topical blood-letting around or in the neighbourhood of the seat of the eruption is always advantageous. All topical applications ought to be of the soothing and relaxing kind.—Tepid bathing, folds of soft linen dipped in simple tepid water and kept constantly applied to the parts affected are extremely soothing. Saline medicines and antimonials are indicated, and the bowels should be kept in a lax condition by the use of one of the aperient neutral salts. Depletion, in one shape or other, and carried, according to circumstances, to a greater or a less extent, is *the* thing for eczema, acute or chronic.

*Herpes Iris* is a rare affection, and many of our readers may never have witnessed it. It is usually observed in the young. Dr. Willis has seen it in two instances. He gives an account of it as it appeared in a female seventy years of age. "The eruption," he says, "was preceded and accompanied by a good deal of tingling and smarting in the parts affected, and by a considerable amount of general constitutional disturbance. It began with circular patches, which showed themselves principally on the palmar, dorsal, and lateral aspects of the fingers, over the joints and knuckles, though some also occurred on the insteps and ankles, and several on and around the elbows. The centres of the patches were occupied by a pale raised wheal, which at first was not vesicular, though it looked as if it contained fluid; this was surrounded by a narrow ring of rather dusky inflammation; which was inclosed, in its turn, by a second also narrow ring of a pale pink or waxen hue, raised like the central spot; a third ring of dusky inflammation surrounded these two, the colour of which was gradually lost in that of the surrounding skin. Although the central spots generally looked vesicular, fluid was only discovered in one of them; the others, at an early period, were certainly solid, and bled when they were pricked; at a later stage they were made up of loose epidermis of considerable thickness that appeared to have been detached by effused fluid. In no instance was the second raised pale coloured ring found to contain fluid. Each spot preserved its characteristic appearance for six or seven days; it then began to desquamate, the cuticle becoming loosened first in the centre, and next over the pale raised ring, but being finally detached from the whole of the inflamed area. The eruption continued during the course of nearly five weeks; as one spot faded and desquamated another made its appearance."

In all the cases of *Cancrum Oris* which Dr. W. has seen, the disease appeared to begin in the gums. These parts, he observes, without having exhibited any great degree of previous swelling, though they may have been red and painful for some time, seem to lose their vitality on the surface and especially along the margins in contact with the necks of the teeth; they appear as if covered with a layer of false membrane, which however is sunk into rather than deposited on them; they look exactly as if they had been changed into so much moist and ragged chamois leather. The living structures in contact with the sphacelated portions are red and painful, and bleed with the slightest touch. There is at the same time a considerably increased secretion of saliva, which dribbles from the mouth, especially while the patient is asleep, and the breath becomes horribly offensive. The teeth now become loose, and unless the disease ceases to make progress of itself, or a check is given to it by the interference of art, the alveolary processes perish from necrosis, and the teeth drop out. The disease at the same time generally extends either inwards, involving the tongue, or more commonly outwards, implicating the inside of the cheek, which then presents a sloughy spot, having the same general appearance as the gums. This is a very serious symptom. A grey or livid or dark coloured purple spot then appears on the cheek externally, which by and by becomes brown or black and then shrivels and dries: the whole thickness of the cheek has been stricken with gangrene; and though the extent of the part so involved may at first be no more than a spangle or split pea would cover, the mischief extends with frightful rapidity; the sphacelated spot on the second day will have the magnitude of a fourpenny or sixpenny piece; on the third, of a frank or shilling, on the fourth, of a half-penny, penny, or even crown-piece, and so on; the mischief extending to the same extent internally, the soft parts losing their connexions with the bones beneath, and the bones themselves becoming affected with necrosis.

Dr. Willis has seen many cases at the Infirmary for Children, and the following treatment he has found very successful. "I have very commonly," he says, "begun the treatment by the exhibition of an emetic of ipecacuanha, followed after a proper interval by a purge of compound powder of jalap and calomel, which it has generally been found necessary to repeat oftener than once, and then by saline medicine with tartrate of antimony. In the milder cases the diluted hydrochloric acid is the topical application upon which I have generally depended, but this has been sometimes replaced by a strong solution of one of the metallic salts, such as sulphate of copper, sulphate of zinc, or nitrate of silver. In the severer forms, where the disease was spreading onwards to the tongue and cheeks, or had already reached these parts, the strong nitric or sulphuric acid becomes the proper application. I have generally prescribed the former. The kind of stimulant or escharotic is not, however, as I imagine, of any great consequence. The disordered actions in the system generally, the tendency of which was to cause the death of some of its parts, having been corrected by the effects of emetics, aperients, and saline and antimonial medicines, a local stimulus of power adequate to insure contraction in the weakened and over-distended capillaries of the structures immediately affected, is all that is necessary. If Peruvian balsam, tincture of myrrh, or pyroligneous acid suffice for this end, there is no necessity to have recourse to the hydrochloric, sulphuric, or nitric acid. A solution of the chloride of sodium, or chloride of lime, is an excellent means not only of correcting fetor of the breath, but of stimulating the diseased parts and disposing them to healthy action, and should always be recommended, even though escharotics of the most active character may be required at the same time. It is often advisable to extract teeth that are shaky, and apply stimulants or escharotics to the sockets." We would observe that Dr. Willis *never* prescribes wine in the diseases of childhood. "Panada with a little new milk; sago or arrow-root sweetened with sugar and seasoned with lemon or orange juice, our ordinary fruits baked or boiled, and sweet oranges, I have always seen to agree, and I think to prove beneficial."

FASCICULUS XI. delineates *Syphilitic Maculæ—Syphilitic Tubercle—Syphilitic Lichen.*

FASCICULUS XII. has another representation of *Syphilitic Tubercle—Syphilitic Pustule—Warty Syphilitic Eruption.*

Fasciculus XIII. has *Syphilitic Verrucæ* on the penis and at the anus—*Syphilitic Lichen* again—*Syphilitic Eczema* desquamating—*Syphilitic Exanthema.*

Fasciculus XIV. has *Psoriasis Inveterata—Eczema Impetiginodes—Impetigo Sparsa Capillitii—Impetigo Sparsa et Conferta Capillitii.*

Fasciculus XV. (the last which has been published,) contains *Lichen Simplex* desquamating—*Ecthyma—Tumor Follicularis*—and *Nævus.*

Speaking of *Follicular Tumor,* (Molluscum?) Dr. Willis observes that, the sebaceous follicles of the skin are subject to a considerable number of affections, which by writers on diseases of the skin have been separated from one another, and referred to very different parts of their nosological charts. We sometimes see these follicles considerably increased in size, assuming a morbid activity, and pouring out such a quantity of sebaceous matter that it dries and concretes on the surface in the guise of a continuous crust of ceruminous matter, which looks extremely like altered epidermis. This is most apt to happen on the face; and a disease of the sebaceous follicles of this part, of the kind described, has actually been described under the title of icthyosis of the face. Again, the secretion of the sebaceous follicles is often altered in quality; in particular, it becomes so thick that it cannot escape on the surface which it is designed to lubricate, but remains pent up in the secreting cavities, and there it either remains quiet and causes what are called Crinones or grubs of the skin; or exciting inflammation in the surrounding dermal tissue, it gives rise to the pustular disease named Acne; or accumulating in larger quantity and producing a kind of hypertrophy in the follicle, an elevation or tumor of the surface is the consequence.

He adds:—"There can be little doubt of the disease which Dr. Bateman described under the name of Molluscum, being due to the distention and induration of the sebaceous follicles. The disease is most common on the face and neck. The tumors which constitute it vary from the size of a small pin's head to that of seed-pearl, or small peas. When attentively examined, these little tumors, which the vulgar often call warts, are found to have a rough and depressed point at one part, which indicates the natural orifice of the follicle, now enlarged and distended. They are of a waxy white colour, sessile or pediculated, and have often a sort of opalescent semi-transparency. It is very common to observe several children of the same family affected with these follicular tumors; and the writer has on two occasions seen the disease to all appearance transferred to the neck and shoulder of the mothers of infants who had many of them upon the face. However unlikely, therefore, the disease would really seem to be contagious." These follicular tumors are commonly seen in early life. They are readily emptied after being punctured with a lancet, and frequently their contents do not again accumulate. They may certainly be prevented from doing so by a due application of caustic to the secreting surface of the cyst. When the tumors are pediculated they are readily and effectually removed by being tied with a fine silk thread.

In concluding this notice of the Fasciculi before us, we cannot refrain from pointing out their general accuracy and pictorial merits. The collection of plates, when complete, will be highly valuable, and the work is richly deserving of encouragement. It must be a matter of regret that its price will put it beyond the reach of many members of the profession, but when we consider the extensive nature of the subject, and the costliness of art in this country, it is only what might be expected. We wish Dr. Willis, a very zealous and a very well-informed physician, every possible success, and we shall not forget to bring the subsequent Fasciculi before our readers as they appear.

THE CYCLOPÆDIA OF PRACTICAL SURGERY, EMBRACING A COMPLETE VIEW OF ALL THE DEPARTMENTS IN OPERATIVE MEDICINE. Edited by *William B. Costello*, M.D. Member of several Learned Societies, National and Foreign. Part V. London, Sherwood and Co.

THE fifth Part of this Cyclopædia arrived too late in the quarter, to be included in the same notice with the other Cyclopædiæ. Our space is too crippled here to allow us to do more than merely enumerate its contents. A further account of them will be found in our next number.

The articles are :—Asphyxia, P. Bennett Lucas, Esq.; Auscultation, Charles Benson, M.D.; Avulsion, J. M'Farlane, M.D.; Balanitis, Henry James Johnson, Esq.; Bandage, H. T. Chapman, Esq.; Bladder, W. B. Costello, M.D.; Blister, Alexander Ure, M.D.; Blood-letting, J. Wardrop, M.D.; Bone, T. Spencer Wells, Esq.; Bougie, W. B. Costello, M.D.; Bronchotomy, T. Spencer Wells, Esq.; Bubo, Henry James Johnson, Esq.; Burns and Scalds, Alexander Ure, M.D.

The Editor has redeemed his promise of publishing the present Part in March, and we trust that the subsequent ones will appear at the appointed times.

---

A TREATISE ON SIPHILIS. By *Herbert Mayo*, F.R.S. Senior Surgeon of Middlesex Hospital, formerly one of the Professors of Anatomy and Surgery to the Royal College of Surgeons. London, Henry Renshaw. 8vo. pp. 128.

MR. MAYO must be well known to all our readers. He is one of the readiest writers in the surgical world. We have been indebted to him for commentaries on Anatomy and Physiology, a Manual of Anatomy, Elements of Physiology, a Manual of Pathology, a Treatise on Diseases of the Rectum, a Treatise on Indigestion, a Series of Letters on Animal Magnetism, and the present work upon Siphilis, besides some papers which may at this moment escape our recollection. Medicine, surgery, physiology, pathology, have been enriched by his observations, which have been always ingenious, frequently acute, but too commonly precipitate. Perhaps it would have been better had Mr. Mayo displayed less facility and more caution. His authority would have been more respected, and his position more stable, than they are at the present moment.

The work before us having already appeared in the shape of detached Essays in a contemporary Journal of extensive circulation,* it is not consistent with the plan we have acted on, to review it or to analyse it. We shall limit ourselves to a few words on the "Conclusions," with which our author winds up the work.

1. "The first point of inquiry relates to the means of discriminating the primary local disorders that are liable to be followed by constitutional lues. I have not thought it necessary among these disorders to enumerate gonorrhœa. Either gonorrhœa never originates lues, or originates it in so few instances, that it would be extravagant to superadd to the treatment of that complaint a preventive course of mercury. The local affections which are certainly liable, but in very different degrees, to be followed by lues, may be divided into three kinds: first, those which are again so rarely followed by lues as to render a preventive course of mercury superfluous; secondly, those in which, though not unfrequently followed by lues, mercury is generally prejudicial; thirdly, those, which are in a large proportion followed by lues, to the speedy cure of which, and to the pre-

* The Medical Gazette.

vention of the lues consequent upon them, mercury has been ascertained to conduce. The first kind includes excoriations, herpes, preputial ulcers, warts; the second, ulcerative and sloughing phagedæna; the third chancre. The diagnosis of chancre is to be obtained in common cases by the direct knowledge of what are the appearances of other sores in their simple state, and what the natural appearances of chancre, and to what extent the features of both may be confused by inflammatory accidents. In ambiguous cases the history must be taken into account. M. Ricord's test by inoculation may likewise be advantageously resorted to. But, as I have stated, that test has less practical than speculative value. Taken alone it is insufficient to decide the question of treatment. A decided mercurialist certainly would not prescribe mercury in many cases of primary sores, inoculation from which will produce an ulcer resembling chancre."

This appears to us to be a fair expression of the facts.

2. The arguments for and against the mercurial treatment of "chancre," (the word were well expunged) are thus stated :—

"The arguments in support of the non-mercurial practice, and against the use of mercury are—that a mercurial course is a serious inconvenience to any one, and that to many constitutions it is most injurious;—that every primary sore may be healed without mercury;—that the most serious form of lues, ulcerative cutaneous disease, namely, is probably not capable of prevention by a mercurial course;—that the other secondary symptoms are, in the majority of cases of non-mercurial treatment, extremely mild;—that they supervene earlier, and are generally sooner over, where a course of mercury has not been used for the primary complaint;—that the secondary symptoms which occur where mercury has not been used, are in no cases more severe than in many in which the mercurial treatment has been followed;—that mercury does not prevent, but only lessens the frequency of, constitutional lues, its direct action apparently being to put off the invasion of lues so long, that in a certain proportion of cases the impression of the taint wears out before it has given rise to secondary symptoms. These considerations are so favourable to the non-mercurial practice, as to render its adoption adviseable in all cases in which, from inherited or accidental peculiarity of habit, mercury is likely to prove injurious; in cases in which the patient has unluckily, through the recurrence of primary sores, undergone several mercurial courses in a short space of time, and is weakened in health; in cases in which the primary sore has already existed a long time, in cases in which there is a reasonable doubt whether the local affecion is syphilitic.

The considerations which, on the other hand, favour the mercurial practice, are, that to persons of a good constitution, a mercurial course, properly managed, very rarely proves detrimental;—that on a comparison of all the evidence, there is reason to believe that the proportion of cases in which secondary symptoms appear after non-mercurial treatment is greater than where mercury has been used;—that it is highly probable that among the attacks of secondary siphilis prevented by mercury, are several of the severest kinds (setting aside the ulcerative);—that if the general tendency of mercury, where it stops short of preventing secondary symptoms, is to protract the whole period of the disease, yet that this is not constant, and that without mercury equally lasting attacks are met with;—that on Mr. Rose's own estimate, the proportion of cases in which secondary symptoms occurred under the non-mercurial practice is much higher than commonly imagined: Mr. Rose observes, that out of one hundred and twenty cases of venereal sores which he had himself treated without mercury, one out of every three was followed by some form or other of constitutional affection; 'but this, however,' he adds, 'was in most instances mild, and sometimes so slight, that it would have escaped notice, had it not been carefully sought after;' and a similarly close scrutiny might detect many overlooked cases of lues after mercurial courses.—Finally, that in cases of indurated chancre, the period of recovery is shortened by the use of mercury ; that the sore is so healed thoroughly and soundly, and that all chance of the local complaint again breaking out is thereby put an end to, and the risk of afterwards unsuspectingly so conveying infection avoided. Weighing these facts against those advanced on the other side, I think it fairly appears, that in a person of good constitution, the treatment of chancre, undertaken soon after its commencement, should include a full mercurial course.

There exists, indeed, a middle plan ; namely, to employ mercury to the extent merely that may be necessary for the cure of the primary sore. By this means several of the advantages of mercurial practice are certainly obtained, and some of its evils avoided. In general, the quantity and duration of mercurial action required to heal primary sores does not amount to that which is supposed to be essential to the prevention of constitutional lues. This plan may be viewed as the most advisable practice in cases of indurated chancre, in which the disease has already gone on for several weeks without any use of mercury."

We are inclined to look on the arguments for a mercurial course as understated. Mr. Rose, himself, is well known, in later life, to have practically turned his back on the non-mercurial treatment, and we can ourselves testify to his extreme want of success from non-mercurial remedies. If we are not much mistaken, Mr. Guthrie, whose earlier cases looked so promising for that method, will not be found to treat the chancres of his private patients without mercury— admissions these, as significant as possible of *some* fallacy *somewhere* in the non-mercurial method. That *that* is inferior to a moderate mercurial one, we are as convinced as we are of any thing in medicine.

Of the "middle plan" of which Mr. Mayo speaks, and to which he seems to lean, we can entertain no very favourable opinion. The great use of mercury is ultimate security. Does *it* give *that?* If a person has not a *fair* course, he had better, in most cases, have none. We have seen these half-and-half mercurial courses tried; the result was not encouraging. We do not see why it should be applicable to an "indurated chancre which has already gone on for several weeks without any use of mercury." Induration and duration are no valid grounds for pushing mercury beyond a reasonable quantity; but neither do they seem to us to be grounds for keeping it down below that.

3. "The symptoms of secondary siphilis are pains, eruptions, and ulcers on the skin, falling off of the hair, soreness and ulceration of the fauces and nose, iritis, swellings of the bones and joints. Either of these may form the only symptom manifested. But in most cases several are grouped together simultaneously or by succession ; and the phenomena of the disease are conveniently displayed by supposing six modes of secondary siphilis, taking as the type of each the feature most developed in it. These six comprise three, in which the skin is the part prominently affected ; one in which the throat, a fifth in which the iris, a sixth in which the bones, are the parts principally concerned. The rules for the treatment of each variety have important shades of difference ; but one principle, with exceptions which I have specified, reigns throughout. The essential principle in the treatment of constitutional lues is to aim at no more than subduing the present attack, keeping the patient during the remissions of the disease in perfect but not rude health by the strictest regimen and course of living, in the expectation that the disease will thus eventually wear itself out. In the choice of remedies to subdue each attack, exclusive of the iritic, mercury is to be shunned, or to be resorted to most seldom. Mercury, with occasional rare exceptions, acts only repellently in secondary siphilis; it does not then extinguish the disease; it only then, like the other antisiphilitic remedies, represses the present outbreak. The fatal character which the disease often wore, so recently as thirty years back, arose from men's constitutions being destroyed by repeated courses of mercury, given in the vain expectation of cutting short the disease when in its constitutional form, and in the still more mischievous belief that nothing but mercury would cure even the present attack; and that the worse the symptoms grew under its administration, the greater became the necessity of continuing it."

This, we think, takes a limited, if not erroneous view, of the treatment of secondary syphilis. But that is too wide a question to make the burthen of a paragraph.

We have presented these "Conclusions," because they give us the gist of the work. It bears the usual impress of Mr. Mayo's pen—rapid, sketchy, more bold in its generalizations than cautious—clear, because perhaps it has not depth—pleasing, from its easy style, if not from its light matter—we perceive in it the author of the Outlines of Physiology.

---

Aphorisms on Insanity. By Dr. *Millingen*. Duodecimo, pp. 202. Churchill, February, 1840.

It was naturally to be expected that, when a ready writer and keen observer, like Dr. Millingen, was thrown into the management of a great lunatic asylum, like Hanwell, he would turn the appointment to advantage, and either make original observations or polish those on record, so as to bear a new and attractive aspect. Dr. M. has done both—and he does a third thing, as important as either—selects the wheat from the chaff of lunatic literature, and in one small pocket volume, has compressed more real and solid matter than could be gleaned out of any dozen of octavos on the same subject. The work is thrown into 503 aphorisms, and as these are conveyed in concentrated language, it is impossible to offer any thing like an analysis of them. All we can do is to quote a few specimens, taken almost indiscriminately, by which the reader may form some estimate of the volume. We may preface these samples, however, by stating that our author makes war on many of the "things that be," not being very sparing of criticism—or even censure, when either "mad doctors," or mad-houses come in his way.

"Thus are the junior members of the medical profession engaged in practice, with scarce any knowledge of the nature of insanity, or the treatment of the insane; while their constant occupations, which barely leave them leisure for such indispensable reading as is required to keep them on a level with the progress of the science, prevent them from studying a disease which they are seldom called upon to attend, and is considered as the special province of those practitioners whom the public dignify by the appellation of '*Mad Doctors;*' and, even amongst these privileged persons, the treatment of mental disease appears to be grounded on certain dogmatic principles; bleeding, blistering, purging, vomiting, and head-shaving, being considered immutable remedial agents, aided by the strait-waistcoat, the sleeve, the muff, the handcuff, or the leg-lock, with an occasional plunge in cold water, or a shower-bath, when the patient is unruly, and sane enough to consider himself most barbarously ill-used. With this view of the subject, we find that in certain lunatic institutions the unfortunate inmates are bled in the month of June, and are prescribed an emetic 'all round' every quarter. The science of 'mad doctors' seems to consist in the art of obtaining as many patients as they can in private asylums, and to keep them as long as possible."

This, we believe, is applicable to a very few of the respectable asylums and their keepers.

Aph. 1. *Definition.*—"Every morbid state that influences our reflective, observant, and imaginative faculties, disables an individual from conducting the process of reasoning, or the sound and healthy exercise of his mental attributes, constitutes insanity."

APH. 5. *Etiology*.—" Experience tends to show, that the first causes of insanity may be traced more frequently, to mental agency reacting upon the physical functions, until symptoms of a peponderance of the physical phenomena prevail. Thus a sudden moral impression of a violent and intense nature will produce a rapid deviation of the mind from its normal course—when physical phenomena, such as the hair turning white in the course of a few hours—the liver becoming diseased—the cerebral organ engorged and inflamed; will be developed with a rapidity scarcely credible."

APH. 21. " Religious delusions are no doubt the occasional cause of insanity, but these ideas are more generally an effect than a cause, and are to be attributed to the gloom and the fears of the melancholic, who are constantly foreboding evil."

APH. 43.—" *Post mortem* examination frequently exhibits morbid alterations of the structure in the brain and its membranes; but we must recollect that during the violent paroxysms of insanity there is a constant determination of blood to the head, similar to that congestion which arises when a man is under the influence of passion. In such a delicate organ it is natural to expect that some morbid appearance will be traced. Repeated intoxication will be followed by similar results: frequent inebriety may therefore be considered as a powerful predisposing cause, by producing increased action so often, as ultimately to lead to a derangement of the mental faculties."

Had our space permitted, we should have adduced several other Aphorisms here; some will be found in another place. In the mean time, we beg to recommend this vade mecum as the best thing of the kind we ever perused.

---

AN ATLAS OF PLATES ILLUSTRATIVE OF THE PRINCIPLES AND PRACTICE OF OBSTETRIC MEDICINE AND SURGERY. With descriptive Letter Press. By *Francis H. Ramsbotham*, M.D. Member of the Royal College of Physicians, &c. Nos. 1, 2, and 3. London: John Churchill, 1840.

DR. RAMSBOTHAM observes, in a brief Preface, that " should any apology be deemed necessary for obtruding on the medical public a new work on obstetric science, it may, perhaps, be furnished by the interest which that department of medicine has acquired of late years, and the attention it now commands from the profession. The numerous valuable publications on the subject that have recently issued from the press, forcibly demonstrate the high position it has attained as a part of medical studies; and it is confidently hoped that the present addition to the stock of obstetric literature, drawn up on a somewhat novel plan, will not be considered altogether superfluous."

The object of the Work is to place in an attractive, concise, and lucid form before the Student, those principles of obstetric science which must engage his earliest attention. It will be completed in Twelve Monthly Numbers. Each Number will contain six finely executed Plates on steel, descriptive letter-press, and wood engravings printed with the text. The price of each Number is one shilling and sixpence, a price extraordinarily low, for the quantum of matter as well as for the mode of execution; for the plates are engraved on steel.

The *first Part* presents to us the Pelvis and Fœtal Head.

The *second Part* depicts distorted pelves, and the method of ascertaining the diameter of the Pelvis.

The *third Part* contains another view of distorted Pelves, and delineations of the External and Internal Organs.

We can speak very favourably both of the letter-press and of the plates, and as a large sale can alone remunerate the publisher, we wish him that large sale which he deserves.

